# Reversible Decerebrate Posture in Hepatic Encephalopathy: Case Report and Literature Review

**DOI:** 10.7759/cureus.21960

**Published:** 2022-02-06

**Authors:** Saria Tasnim, Randa Hazam, Dhara Dave, Hina Yousuf, Muhammad Khan, Manish Patel

**Affiliations:** 1 Internal Medicine, Texas Tech University Health Sciences Center, Amarillo, USA; 2 Internal Medicine, University of Texas Health Science Center at Houston, Houston, USA; 3 Pulmonary and Critical Care Medicine, Texas Tech University Health Sciences Center, Lubbock, USA

**Keywords:** trans-hepatic portosystemic shunt, liver cirrhosis, trans-hepatic porto-systemic shunt, coma, decerebrate posturing, hepatic encephalopathy

## Abstract

Decerebrate posturing is commonly associated with a global brain insult as in massive strokes, brain stem infarction, or hypoxic brain injury. The clinical presentation of hepatic encephalopathy is variable. ranging from an abnormal sleep pattern to somnolence and deep coma. Decerebrate or decorticate posturing is a rare manifestation of hepatic encephalopathy. We report a patient with liver cirrhosis who presented with a mild degree of hepatic encephalopathy that rapidly progressed to coma with decerebrate posturing. Although the pathophysiology of hepatic encephalopathy is unknown, it appears to be reversible as in our patient who completely recovered with the management of encephalopathy. Despite being uncommon, it should be considered in patients presenting with hepatic encephalopathy progressing to coma since it might save unnecessary workup.

## Introduction

Hepatic encephalopathy describes a reversible syndrome of neuro-psychiatric dysfunction associated with liver dysfunction and/or portosystemic shunting due to impaired removal of neurotoxic substances. It affects approximately 30-45% of patients with cirrhosis and 10-50% of patients with a transjugular intrahepatic portosystemic shunt. The development of hepatic encephalopathy can be spontaneous or precipitated by other conditions, such as infection, sedating medications, volume depletion, electrolyte abnormalities, or gastrointestinal bleeding, and heralds the worsening of liver function. Its presentation can vary from minimal changes like alteration in sleep patterns to personality changes to severe morbidity in the form of frank coma. Posturing usually represents advanced and irreversible damage to the brain, however, it has been known to be an extremely rare manifestation of hepatic encephalopathy with only a few cases previously reported in the literature to date [1]. Here we present one such patient who had reversible decerebrate posturing secondary to hepatic encephalopathy.

## Case presentation

**Figure 1:**
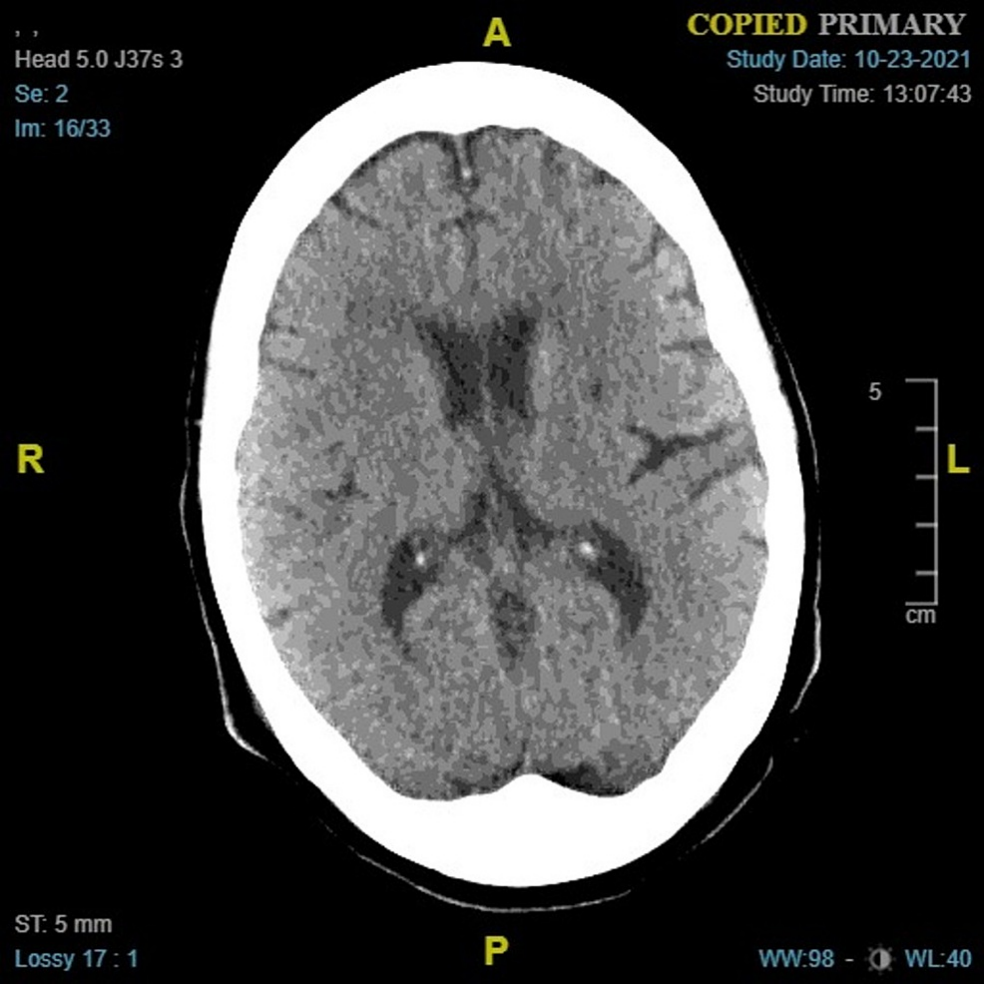
CT head of the patient with no acute intracranial process

A 69-year-old female, with a history of non-alcoholic liver cirrhosis Child-Pugh B with portal hypertension status post-trans-hepatic portosystemic shunt (TIPS) two months prior, was brought by emergency medical service (EMS) with altered mental status. She had a slowed speech and thought process. There were no seizures, weakness, numbness, fever, or chills reported. She has no history of alcohol abuse, tobacco, or recreational drug use. EMS reported blood glucose of 47 mg/dL upon arrival; it improved to 175 mg/dL with D50 administration. On examination, the temperature was 37.5 C, oxygen saturation was 95% on room air, respiratory rate was 15 per min, heart rate was 100 beats per minute, and blood pressure was 130/85 mmHg. On general exam, no asterixis was found, bruises were seen around the left shoulder and anterior abdominal wall. The central nervous system exam was non-focal, Glasgow Coma Scale (GCS) was 15, and the pupils were round and reactive. Labs showed ammonia level 0.212 mmol/L (0.011 - 0.032 mmol/L), white blood cells 9.4 × 103/µL, alkaline phosphatase 77 units/L, aspartate aminotransferase 108 units/L, alanine aminotransferase 36 units/L, thyroid-stimulating hormone 2.5 mU/L, and free thyroxine 0.9 ng/dL. Electrocardiogram and arterial blood gases were unremarkable. CT head (Figure 1) and surveillance X-rays were normal. She was admitted and treated for mild hepatic encephalopathy with lactulose and rifaximin.

Overnight, she became poorly responsive, GCS dropped to 7, and she exhibited generalized stiffness with decerebrate posturing. She was intubated for airway protection. Table 1 shows repeated labs; ammonia level went up to 0.6 mmol/L, blood glucose 149, and urine toxicology was negative. Repeat CT head showed no acute changes, chest X-ray was unremarkable, and MRI showed no territorial infarction or mass-effect. Ultrasound duplex of the TIPS showed a patent stent with normal velocities. She was aggressively treated with lactulose and rifaximin and maintained a good number of bowel movements. Her mentation improved significantly by the second day, with a resolution of decerebrate posturing. She was extubated on Day 3. A detailed neurological exam showed no focal deficit.

**Table 1:** Table showing comparative changes of labs between Days 0 and 2 of admission, indicating no other contributing factors responsible for the acute deterioration of mentation other than hepatic encephalopathy

Lab parameter	Normal Reference Range	Day 0	Day 2
Albumin gm/dl	3.4-5.0	3.0	3.3
Creatine Kinase (CK) units/L	25-232	3466	296
Ammonia mmol/L	0.011 - 0.032	0.212	0.619
International Normalized Ratio (INR)	0.89-1.07	1.44	1.77
Alkaline Phosphatase (ALP) units/L	50-136	77	67
Aspartate Aminotransferase (AST) units/L	15-37	108	22
Alanine Transferase (ALT) units/L	16-63	36	28
Glucose mg/dl	74-106	128	149
Sodium mmol/L	136-145	143	149
Potassium mmol/L	3.5-5.1	3.3	4
Bicarbonate mmol/L	21-32	20	17
Chloride mmol/L	98-107	114	121
Anion gap mmol/L	9-18	9	11
Blood Urea Nitrogen (BUN) mg/dl	7-18	15	19
Creatinine mg/dl	0.6-1.3	0.6	1
White Blood Cell e3/mcL	4.0-10.6	9.4	6.9
Hemoglobin gm/dl	12.0-16.0	11.5	9.2
Mean Corpuscular Volume (MCV) Femtoliters	81-98	88	90
Platelet 10 e3/mcL	150-400	277	207

## Discussion

Hepatic encephalopathy is a common manifestation of patients with decompensated liver disease. It is generally fully reversible without any residual brain damage with aggressive treatment in a timely manner. As common as this condition is, the exact pathophysiology is complex and not fully understood. Theories suggest the implication of elevated ammonia levels, accumulation of false neurotransmitters like octopamine in the brain, oxidative stress, abnormal histamine, and serotonin neurotransmission, enhancement of GABAergic tone, and manganese deposition in the basal ganglia [2].

Presentation of hepatic encephalopathy varies widely from mild lack of awareness, lethargy, somnolence, to hepatic coma. Decerebrate posturing, although rare, has been reported in some patients with hepatic encephalopathy [3-5]. It is described as an involuntary extension, adduction, and pronation of the upper extremities in response to external stimuli. It is usually a manifestation of dysfunction below the red nucleus. Decorticate posturing, on the other hand, is upper-extremity adduction and flexion at the elbows, wrists, and fingers. This occurs with dysfunction at the cerebral cortical level or below. The presence of these is associated with irreversible injuries and implies a detrimental state; however, in most patients with hepatic encephalopathy, the condition was reversible [6].

Decerebrate and decorticate posturing are indicative of advanced and irreversible insults to the brain that can result from structural anomalies like abscesses, intracranial bleeds, infracts, or tumors, traumatic injury to the brain, or obstructive lesions leading to hydrocephalus and/or increased intracranial pressure. Metabolic derangements may also present as posturing in the absence of structural anomalies, for example, infections like meningitis, or encephalitis, electrolyte anomalies like hyponatremia, hypocalcemia, or hypoglycemia, lead poisoning, hypoxic brain injury, and hepatic encephalopathy [6].

The pathophysiology behind posturing in patients with hepatic coma and why some patients develop it while others do not is unknown. Cerebral edema is one of the hypothesized pathophysiologies along with impaired hepatic removal of neurotoxic substances leading to astrocyte swelling and increased formation of reactive oxygen and nitrogen radicals causing inflammation [7]. Given that most autopsies of patients with hepatic coma showed no structural anomalies, Shorey et al. postulated that the neurophysiological basis of coma and other features of hepatic encephalopathy may be due to selective effects of ammonia on the brain stem [8].

As noted, our patient did have an elevated ammonia level upon admission, and this is the most acknowledged theory behind the pathogenesis of hepatic encephalopathy. An extensive search for more common causes of coma with abnormal posturing was unrevealing. Despite the low blood sugar at initial presentation, which was appropriately corrected and remained normal thereafter, her mental state continued to deteriorate, ruling out hypoglycemia as a potential cause. Of note also, the rapid decline in mentation occurred hours after hypoglycemia was managed. Our patient responded well to appropriate management with lactulose and rifaximin and showed a quick recovery within three days.

Despite the poor prognosis associated with posturing due to other etiologies and no specific treatment for posturing, our patient improved nearly completely after 72 hours of supportive treatment, further confirming a transient brain insult that appears to be related to hepatic encephalopathy.

As shown in Table 2, a review of similar case reports via a search on PubMed and Google Scholar, over the past 30 years, showed that all patients were more than 50 years of age, and most had alcohol-related cirrhosis. The most common risk factor predisposing to decompensation was a gastrointestinal (GI) bleed, followed by the presence of a portacaval shunt. It was noted that the development of coma and posturing was usually rapid from the time of development of altered mental status. Compared to earlier cases [3-4], the recently documented ones ([5] and ours) have a much shorter hospital stay and rapid recovery suggesting improved hepatic encephalopathy management strategies. Most patients showed quick recovery and the posturing was completely reversed in those that recovered.

**Table 2:** Patients presenting with hepatic encephalopathy, their onset of neurological symptoms, and their outcomes GI: gastrointestinal; TIPS: trans-hepatic portosystemic shunt

Author (Reference)	Age, Gender	Cirrhosis cause	Risk Factors	Clinical presentation on admission	Timing from admission to coma	Treatment	Outcome
Ish et al (3)	56, M	Alcohol-related	GI bleed	Hematemesis, Altered mental status	On admission	Mannitol, neomycin, evacuation enemas, and sodium cephalothin	Died
Ish et al (3)	60, M	Alcohol-related	GI bleed	Hematemesis	Emergency shunt on admission and immediate postoperative coma	IV fluids, neomycin, low-protein diet	Improved
Conomy et al (4)	51, F	Alcohol-related	GI bleed	Hematemesis	Emergency shunt on admission followed by coma in 24 hrs	IV fluids, neomycin in GI tract, arginine glutamate infusions, evacuation enemas	Improved initially though passed away eventually
Conomy et al (4)	51, M	Alcohol-related	GI bleed	Altered mental status, Hematemesis	Less than 24 hrs	Medical management	Improved
Conomy et al (4)	62, F	Alcohol-related	Portocaval shunt	Unresponsive	On admission	IV fluids, neomycin in GI tract, arginine glutamate infusions, evacuation enemas	Improved
Wehbe et al (5)	59, M	Alcohol-related	TIPS	Unresponsive	On admission	Lactulose, flumazenil	Improved
Our patient	69, F	Non-alcoholic	TIPS	Altered mental status	Less than 24 hrs	Lactulose, diuretics	Improved

## Conclusions

Although hepatic encephalopathy has a plethora of presentations, decerebrate posturing is one of the rarest findings. As a result, often, it is not linked to the diagnosis of hepatic encephalopathy, leading to unnecessary investigations to find out the root cause. Although the finding of decerebrate posturing often leads to a consideration of irreversible conditions, its presence alongside hepatic encephalopathy should prompt us to consider it as a potentially reversible finding that may leave the patient without any residual damage. Clinicians should be aware of this condition in patients presenting with hepatic encephalopathy progressing to coma since it might save unnecessary investigations and reduce cost as well as prevent inappropriate decisions regarding goals of care.
